# A design of a DICOM‐RT‐based tool box for nonrigid 4D dose calculation

**DOI:** 10.1120/jacmp.v17i2.5935

**Published:** 2016-03-08

**Authors:** Victy Y. W. Wong, Colin R. Baker, T. W. Leung, Stewart Y. Tung

**Affiliations:** ^1^ Department of Clinical Oncology Queen Mary Hospital Hong Kong; ^2^ Physics Department Clatterbridge Cancer Centre NHS Foundation Trust Wirral UK; ^3^ Department of Clinical Oncology Tuen Mun Hospital Hong Kong

**Keywords:** deformable dose registration, nonridged 4D dose calculation, DICOM‐RT‐based tool box, 4D RT planning, CA lung

## Abstract

The study was aimed to introduce a design of a DICOM‐RT‐based tool box to facilitate 4D dose calculation based on deformable voxel‐dose registration. The computational structure and the calculation algorithm of the tool box were explicitly discussed in the study. The tool box was written in MATLAB in conjunction with CERR. It consists of five main functions which allow a) importation of DICOM‐RT‐based 3D dose plan, b) deformable image registration, c) tracking voxel doses along breathing cycle, d) presentation of temporal dose distribution at different time phase, and e) derivation of 4D dose. The efficacy of using the tool box for clinical application had been verified with nine clinical cases on retrospective‐study basis. The logistic and the robustness of the tool box were tested with 27 applications and the results were shown successful with no computational errors encountered. In the study, the accumulated dose coverage as a function of planning CT taken at end‐inhale, end‐exhale, and mean tumor position were assessed. The results indicated that the majority of the cases (67%) achieved maximum target coverage, while the planning CT was taken at the temporal mean tumor position and 56% at the end‐exhale position. The comparable results to the literature imply that the studied tool box can be reliable for 4D dose calculation. The authors suggest that, with proper application, 4D dose calculation using deformable registration can provide better dose evaluation for treatment with moving target.

PACS number(s): 87.55.kh

## I. INTRODUCTION

Lung cancer remains the leading cause of cancer‐related mortality. Conventional radiotherapy for early‐stage non‐small cell lung cancer (NSCLC) with or without concurrent chemotherapy is associated with the cancer specific survival rate of below 40% at five years.^1^, ^2^ Dose escalation and hyprofractional dose delivery have demonstrated an improvement in local control and patients’ survival.^3^, ^4^ However, the substantial breathing‐induced tumor motion limits their applications. Studies have reported that up to 40% of lung tumors move by more than 5 mm, of those 10% to 12% move more than 1 cm.^5^, ^6^ Since conventional radiation therapy accounted for no tumor motion during treatment simulation, the planning target volume (PTV) thus formed with extensive margin to avoid geometrical misses of the target. This results in excessive lung tissue irradiation and restricts the ability of dose escalation. In attempt to attain more focusing irradiation to a moving target, several sophisticated treatment delivery methods such as gating, tracking or tumor immobilization with breath‐hold have been developed.^7^, ^8^, ^9^ Those advanced delivery techniques, however, require patients’ compliance with the equipment to achieve the desired outcomes; otherwise the results could be adversely affected.^(10)^

The most consistent and stable tumor movement is expected with natural breathing of the patient. Therefore, despite the availability of the advanced delivery techniques, recent studies have re‐employed the free‐breathing technique with the treatment margin carefully determined to account for the tumor motion. The concept of internal target volume (ITV) to account for geometric uncertainties due to tumor movement has been suggested by the International Commission on Radiation Units and Measurements (ICRU) Report 62.^(11)^ The ITV is defined with the combination of clinical target volumes (CTV) delineated on each of the binned phases of the four‐dimensional computed tomography (4D CT) image dateset. Since the microscopic region of CTV is not visualized, to make the determination of the ITV more applicable, studies^(12)^ introduced the internal gross tumor volume (IGTV), which combined the GTVs contoured on each binned phase of the 4D CT dataset. The ITV was then defined by the IGTV plus a margin that accounts for microscopic disease. Both concepts of ITV and IGTV account for the dosimetric effect in tumor motion from the geometrical aspect but not the temporal: this leads to the potential delivery of excessive irradiation to the neighboring healthy tissue. For instance, tumor coverage shown on each phase of the respiratory cycle occurs during only a fraction of the time, but according to the definition of ITV/IGTV, radiation dose is assigned over the whole breathing cycle. Recent studies^13^, ^14^ proposed to use computation modeling for 4D dose calculation, in which static dose distribution was convoluted with the probability distribution function of organ motion to evaluate the resultant temporal‐spatial dose distribution over the moving target. However, the possible change of the target dose volume due to deformable anatomical displacement (i.e., nonrigid anatomical change) was not attended with this later technique.

To obtain the resultant dose on the effect of spatial and temporal movement, as well as to account for the possible deformable anatomical change, the accumulated dose distribution of a moving target could be evaluated based on deformable image registration (DIR). The DIR tracked the displacement of each voxel of the CT image during a respiratory cycle, the anatomical changes in shape, volume, position, and density during respiration being taken into account. Summation of the dose along the trajectory of each voxel provides the resultant 4D dose. Since the 4D dose calculation is complex and involves considerable quantities of dose data, a DICOM‐RT‐based tool box would be desirable for performing automated dose calculation. A few commercially available software products such as MIM Maestro (MIM Software Inc., Cleveland, OH)^(15)^ facilitate adaptive dose calculation in which the voxel dose is traced between two different time phases based on DIR. Yang et al.^(16)^ recently developed an open‐source software tool called DIRART, which is also used for deformable image registration and adaptive radiation therapy calculation. However, with this software users might be limited by the preselected DIR algorithms. Moreover, data of deformable vector fields are usually concealed within the software so that further validation for end‐users may not be possible. In this study, a computational tool box was designed to enable accumulated dose calculation of a moving target in a complete periodic cycle. The tool box facilitates dose plan transformation from 3D‐to 4D‐based. Compared to the currently available software, the studied tool box allows any kind of DIR algorithm to be implemented for 4D dose calculation with the coordinate‐based deformation vector field provided. Moreover, the tool box was designed specifically for 4D dose calculation in which deformable dose calculation is performed with images obtained in multiple time phases (usually 10 phases), instead of 2 phases dose transformation applied in adaptive radiation therapy. The computational structure and the dose calculation algorithm of the tool box are presented in this study. The advantages of the special structural arrangement of the tool box that permits flexible data manipulation for utilization is also discussed in the latter section. To examine the efficacy of the 4D tool box for clinical applications, the 4D dose accumulation was retrospectively computed in nine clinical cases of lung cancer.

## II. MATERIALS AND METHODS

### A. A design of nonrigid 4D dose calculation tool box

A tool box that consists of five main functions was developed for this study. This was written in the MATLAB (Version 7.6, R2008a, MathWorks, Inc., Natick, MA) scientific software environment, in conjunction with CERR,^(17)^ an open source computational environment for radiotherapy research for DICOM images and DICOM‐RT data access and analysis. The 4D dose calculation was performed using Image Morphing (IM),^(18)^ a deformable image registration tool provided by Brainlab (Brainlab AG, Munich, Germany). This allows the displacement of each voxel to be tracked during a respiratory cycle and consequently the accumulated dose along the trajectory of each voxel to be generated.

#### A.1 Treatment planning data import

**Figure 1:**
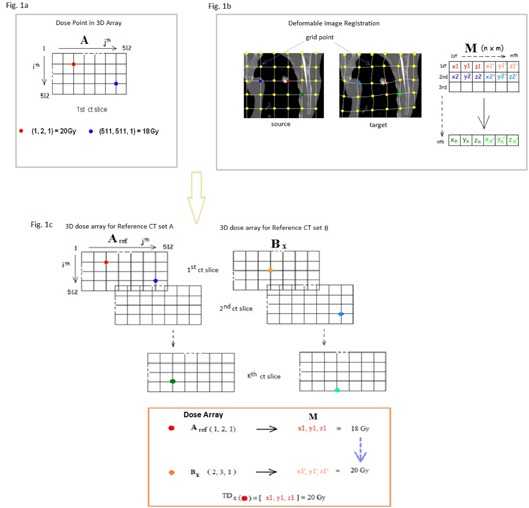
DE‐DOSE‐REG function: (a) description of 3D array dose point in CERR; (b) the morphing process (M denotes the deformation matrix); (c) combination of the 3D dose array and M matrix for 4D dose calculation.

A full set of 4D CT images and the dose plans in DICOM‐RT format were imported into CERR where the dose value and its corresponding position were contained in a 3D array. The positions of the dose points in the transverse plane were defined according to the CT pixels in a 512×512 matrix (Fig. 1(a)). The position along the craniocaudal direction was indicated by the CT slice number. For example A (1, 2, 1) indicates dose point located at the 1st row, 2nd column, and 1st CT slice. The spatial dose resolution (grid interval) defined by the pixel size and the size of the scanning field was 1.01 mm in the lateral and the anterioposterior directions and 3 mm in the caudal‐cranial direction as determined by the CT slice thickness.

#### A.2 Deformable image registration — image morphing

To identify the displacement trajectory of each voxel during breathing, the deformable image registration tools, image morphing (IM) provided by the iPlan (Brainlab AG) treatment planning system was used. IM employs a knowledge‐based segmentation approach^(19)^ in which the anatomical structures are morphed from the source images to the target images by comparing the image data. The source data are deformed based on similarity calculation using mutual information and cross‐correlation algorithm to improve the similarities in voxel density until a match is found. Once the similarity measure is defined, the registration algorithm optimises the similarities by adjusting the transformation vectors in an iterative process. When the point‐to‐point correspondence of the two datasets is identified, the software automatically transfers all outlined structures of the source onto the target dataset. As a result, the deformation vector field, which defines a unique mapping from the source onto the target data set, is generated. Figure 1(b) illustrates the morphing process: IM creates a virtual grid of points in the source data set and matches them with a grid of points on the target dataset. Since each grid point is indexed and its location is recorded in 3D coordinates, the voxel displacement caused by breathing can be traced by the position of the corresponding grid point. The output file generated by IM is formatted into a 6D array with the 3D CT coordinates of the corresponding grid point for the source data set and the target dataset recorded in the 1st to 3rd components and 4th to 6th components of the array, respectively. For example, M denotes the morph array, and M(1)=[1,2,3,4,5,6] implies that the first voxel (or grid point) with the CT coordinates of (1, 2, 3) in the source image was displaced to the CT coordinates of (4, 5, 6) in the target image (Fig. 1(b)). The 3D spatial resolution of the grid point was 1 mm.

#### A.3 DE‐DOSE‐REG function

The function of DE‐DOSE‐REG is to register each voxel of the reference CT with the temporal dose received by the corresponding voxel of each individual 4D CT set. The corresponding voxels were acquired with the morphed vectors contained in the M matrix (n×m) generated by IM, where n denotes the number of voxels and m their coordinates. In accordance with the displaced position of the voxel, the temporal dose at each voxel was traced with the 3D dose array (Fig. 1(a)) obtained from the individual 4D CT plans. In order to match the positioning format between the 3D dose array and the M matrix, the 3D dose array containing dose points in grid order was converted into 3D CT coordinates. Moreover, the spatial dose resolution along the craniocaudal direction, which was defined by the slice thickness, was refined from 3 mm to 1 mm by linear interpolation between point doses. The temporal doses at each bin of phase‐sorted 4D CT images were finally stored in a 1D dose array, namely ${\text{TD}}_{X}$ (where x denotes the phase bin; the percentile of the period of the corresponding breathing cycle of the patient), in the order of voxels presented in the source data of M matrix (i.e., $M_{1-n},{\;}_{1-3}$). The procedures for determining the DE‐DOSE‐REG function are illustrated in Fig. 1(c).

#### A.4 AUTO‐CERR‐DOSE function

**Figure 2:**
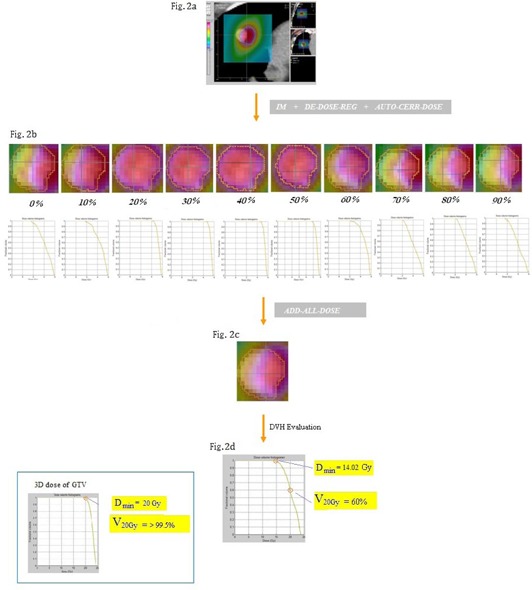
An example of 4D dose calculation: (a) dose distribution shown at end‐inhale phase with the planning CT taken at the end‐exhale phase; (b) target doses received at different phases and the corresponding DVHs; (c) accumulated target dose for a complete respiratory cycle; (d) the DVH of the accumulated target dose.

This function permits the temporal dose received by each voxel at each respiratory phase to be revealed visually on the reference CT. This is done by replacing the 3D dose array of the reference plan with ${\text{TD}}_{X}$, and superimposing the new doses on the reference CT (Fig. 2(b)).

#### A.5 ADD‐ALL‐DOSE function

**Figure 3:**
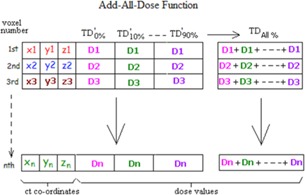
${\text{TD}}_{X\%}^{\prime }$ denotes dose received at the x% breathing phase by multiplying a time weighting factor to ${\text{TD}}_{X}$.

**Figure 4:**
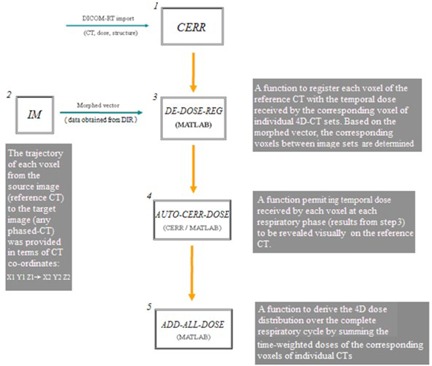
A flowchart of the 4D tool box.

This function derives the complete 4D dose distribution over the entire respiratory cycle by the time‐weighted dose summation of ${\text{TD}}_{X}$ for all phases (Fig. 3). Together with the function AUTO‐CERR‐DOSE, the resultant 4D dose is displayed and assessed geometrically under the CERR environment. The complete process of 4D dose calculation using the tool box is illustrated in Fig. 4.

### B. Evaluation of deformable image registration, IM

The performance of image morphing using IM was evaluated by calculating the Matching Index.^(20)^ The Matching Index is defined as the ratio of the intersected volume between the morphed contour and the manual contour to the union of the two volumes. Using CT lung window, the GTV was manually delineated and the lung volume using autosegmentation (iPlan) with manual correction where necessary. The contours were initially delineated on the planning CT selected at end‐inhale (${\text{PLCT}}_{\text{in}}$) as the source contours and were morphed onto the planning CT selected at end‐exhale (${\text{PLCT}}_{\text{ex}}$) defining the morph_contour, which was then compared with the contours manually delineated on the ${\text{PLCT}}_{\text{ex}}$. The contouring was performed by a physicist, and confirmed by an oncologist if any uncertainty was found.

(1) $$Matching\;Index=\frac{morph\_contour\cap manual\_contour}{morph\_contour\cup manual\_contour}$$

### C. 4D dose study on clinical cases

#### C.1 Case selection

From 2008 to 2010, 10 patients who were diagnosed with stage 1 lung cancer underwent 4D CT simulation for hypofractionated radiation therapy in Tuen Mun Hospital (TMH), Hong Kong. 4D CT imaging was applied to assess the tumor movement and consequently to process 4D dose calculation based on DIR. Stable and consistent breathing is a prerequisite for the control of qualitative reconstruction of 4D CT images, which also substantially affects the DIR performance. In this study, cases with low‐noise and artifact‐free (induced by abrupt breathing) images were selected according to the breathing regularity. To assess the regularity of the breathing rate, the mean breathing rate and the standard deviation (SD) were calculated. Cases with variation of breathing rate (i.e., the percentage SD from mean breathing rate) less than 10% would be selected for the study. Two additional cases (cases 8 and 9) were provided by the Clatterbridge Cancer Centre (CCC), UK. To ensure a regular breathing pattern, patients of CCC group were coached during CT scanning. All studied cases were chosen with well‐defined GTV, which was usually the case with adenocarcinoma confirmed by histology.

#### C.2 Implementation of 4D dose calculation

In principle, the 4D dose over the tumor volume can be calculated by converting the 3D spatial dose to the 4D spatial‐temporal dose. With 4D dose planning, the receiving dose by a moving target could be evaluated by summing the 3D spatial dose of the planning target volume recorded at various time phases. Such a dose‐calculation algorithm was exercised in this study. The 4D spatiotemporal dose was retrospectively calculated in nine clinical cases with the 4D tool box. In clinical application, dose prescription is usually applied to planning target volume (PTV), which is defined with the target volume plus the setup margin. Since the accuracy of IM for deformable image registration was verified by comparing the volumes of the planning targets before and after image deformation, the GTV which can be visually localized on the CT images was therefore employed as the planning target volume in the study. The GTV was manually delineated using a lung window (500‐1000 Hounsfield units). To process 4D dose calculation, the 3D dose planning was performed on the reference CT selected from the 4D CT images. In the study, the 4D CT was acquired with a Philips Brilliance multislice CT scanner (Philips Healthcare, Andover, MA). The respiratory phase was tracked using an infrared optical system (RPM system, Varian Medical Systems, Palo Alto, CA). An infrared marker was placed on the xiphoid of the patient for respiratory tracking. A spiral scan was performed to cover the whole lungs. The scan was acquired at a sufficiently low table speed (low pitch) for any scanned voxel to remain within the detector collimation throughout the complete breathing cycle. The source data and the respiratory signal were retrospectively sorted by phase and reconstructed to produce the 4D CT images. Each 4D CT dataset was comprised of 10 3D CT images spaced equally among the respiratory cycle. Conformal dose plan was obtained with single or multiple coplanar conformal arc/arcs delivered with micromultileaf collimators (M3, Brainlab AG). Dose planning was performed with a prescription dose of 20 Gy at 85% isodose to enclose ≥99.5% of the planning target volume. To process 4D dose calculation, the 3D dose plan at each respiratory phase was recalculated by reapplying the planning parameters and the monitor units obtained from the reference plan to each of the 4D CT bins. The complete set of 3D dose data was imported into the tool box for 4D dose processing as described in the previous sections.

The procedure for 4D dose calculation is illustrated in Fig. 2. An example is shown with the planning CT taken at the end‐exhale phase (50%). Figure 2(a) shows the 3D dose distribution obtained at the end‐inhale phase (0%), the blue contour indicates the original location of the GTV captured on the reference CT (50%). The large discrepancy in location between the dose color‐wash and the GTV shown on the CT image clearly demonstrates the effect of tumor motion on the accumulated dose. Using the functions IM, DE‐DOSE‐REG, and AUTO‐CERR‐DOSE, the 4D doses acquired at each individual temporal phase from 0% to 90% $\left({\text{TD}}_{0\%-90\%}\right)$ and the GTV dose‐volume histogram of each are shown in Fig. 2(b). The accumulated dose over the tumor in a complete respiratory cycle is presented in Fig. 2(c). Figure 2(d) demonstrates the difference on dose coverage of GTV between the original 3D dose plan and the resultant 4D dose using dose volume histogram. Due to the significant tumor movement, the $V_{20\text{Gy}}$, which denotes the amount of target dose coverage at 20 Gy, was 60% of the GTV volume. And the minimum dose ($D_{\text{min}}$) received by the GTV was 70% (14.02 Gy) of the original planning dose (20 Gy).

#### C.3 Target dose coverage as a function of planning CT

The difference in accumulated dose coverage as a function of the planning CT taken at different breathing phase was studied. The $V_{20\text{Gy}}$ was evaluated with the planning CT taken at end‐exhale (50%), end‐inhale phase (0%) and the temporal‐mean tumor position for all cases. Twenty‐seven applications were applied altogether, in which the logistics and robustness of the tool box for 4D dose calculations were examined.

To enable unbiased comparison of the dose studies, similar dose planning parameters were attempted for all plans of each study. Since the shape, size, and location of the planning target were not the same because of the nonrigid transformation of the tumor during the breathing cycle, the planning parameters (e.g., beam aperture margin, number of conformal arcs) inevitably had to vary in order to achieve the optimal dose plan for each study. Similarity in dose properties between the studied dose plans was however confirmed by employing the following dosimetric indices: target coverage index (TCI), conformity index (CI), and dose heterogeneity index (HI).^21^, ^22^ The TCI specifies the percentage coverage of the prescription dose, whereas the CI indicates the amount of prescription dose volume as a function of the planning target. The HI defines the ratio of the maximum target dose to the prescription dose. Since the target dose distribution in terms of dose conformity and dose heterogeneity could be affected by the beam aperture, similar aperture margins ranging from 1.0‐1.5 mm (a larger margin would be required for a less‐regular‐shaped target) were applied for all cases. The pencil beam algorithm implemented in the iPlan planning system (v.4.1.2, Brainlab AG) was applied for dose calculation. An algorithm that accounts for lateral electronic disequilibrium would be preferable, but was not available at the time of this study. However, since dose plans for comparison were performed under the same conditions, the influence of lateral electronic disequilibrium on the radiation planning using pencil beam between the dose plans was considered to be comparable.

## III. RESULTS

The disease characteristics in terms of tumor size, location, and magnitude of movement are listed in Table 1. The GTV volumes were defined in different breathing phases (i.e., inhale, exhale, and mean tumor position). The mean volumes ranged from 1.42 cc to 17.89 cc (2nd column, Table 1) were shown to have small standard deviations (SD) in all cases. The resultant tumor movements defined by the quadratic sum of the maximum movement along the three axes ranged from 1.2‐10.5 mm. Cases with variation of breathing rate over the period of CT scan ranged from 6.1%‐9.7% were included in the study (4th column, Table 1).

**Table 1:** The disease characteristics

*Patient*	*Gross Tumor Volume* mean±SD *(cc)*	*Location*	*Breathing rate variation* $\frac{\text{STD}}{\text{Mean}}x100\%$	*Tumor Displacement (pk‐pk) (mm)*
1	1.42±0.07	LUL	3.4%	5.8 (LAT) 1.6 (AP) 0.3 (CC) 6.0 (RES)
2	1.93±0.06	LUL	9.5%	0.5 (LAT) 2.6 (AP) 3.5 (CC) 4.4 (RES)
3	3.00±0.14	RLL	9.8%	0.5 (LAT) 2.6 (AP) 3.0 (CC) 4.0 (RES)
4	6.74±0.19	LUL	8.5%	1.1 (LAT) 0.5 (AP) 0 (CC) 1.2 (RES)
5	4.06±0.18	RUL	6.4%	1.1 (LAT) 3.2 (AP) 3.0 (CC) 4.5 (RES)
6	17.89±0.33	RLL	9.7%	2.1(LAT) 1.6 (AP) 6.0 (CC) 6.6 (RES)
7	12.80±0.54	LUL	3.3%	1.5(LAT) 3.5 (AP) 0 (CC) 3.8 (RES)
8	5.5±0.47	LUL	unknown	1.5 (LAT) 1.5 (AP) 9.0 (CC) 9.2 (RES)
9	$12.40^{'}\pm 1.47$	LLL	unknown	2.4(LAT) 8.3 (AP) 9.2 (CC) 10.5 (RES)

LUL=left upper lobe; RLL=right lower lobe; RUL=right upper lobe; LLL=left lower lobe; LAT=lateral; AP=anterioposterior; CC=craniocaudal; RES=resultant displacement.

### A. Evaluation of deformable image registration of IM

The matching indices between the manual_contour and the morph_contour of lung volume and of the GTV for all cases were found to be between 0.93 and 0.98 (mean=0.95±0.01) and between 0.74 and 0.98 (mean=0.84±0.08), respectively. The index values are listed in Table 2. The matching indices for lung mapping were comparable with those (range: 0.94‐0.95) reported in the study by Lin et al..^(20)^ The lower matching index for the GTV may be due to the limitation of image spatial and contrast resolution which would affect manual contouring and as well as the results of morphing when the tumor volume is small.

**Table 2:** Matching indices between the manual_contour and the morph_contour of lung volume and of the GTV for all cases

	*Matching Indices manual_contour* (${\text{PLCT}}_{\text{ex}}$) *vs. morph_contour* $\left({\text{PLCT}}_{\text{in}}\to {\text{PLCT}}_{\text{ex}}\right)$	
	*GTV*	*Lung*
1	0.79	0.95
2	0.81	0.98
3	0.74	0.96
4	0.94	0.93
5	0.75	0.95
6	0.94	0.93
7	0.90	0.95
8	0.84	0.96
9	0.98	0.96
Mean±SD	0.84±0.08	0.95±0.01

### B. Dose coverage as a function of planning CT

For dosimetry comparison between dose plans, the similarity in dose properties among the three phase‐specific dose plans was verified with the dosimetric indices. The mean and standard deviation of CI, HI, and TCI of the three phase‐specific dose plans of all cases are listed in columns 2‐4 of Table 3. The small standard deviation of the TCI indicates that the target coverages between the studied dose plans were comparable. In addition to equivalent target coverage, the small standard deviations of CI and HI were 2%‐3% and 0%‐1% of their respective mean values, suggesting that the dose properties and the dose distributions were similar among all dose plans.

The percentage of target dose coverage $V_{20\text{Gy}}$ as a function of phase‐specific planning CT for all cases was calculated using the tool box. The results are listed in Table 3. The maximum dose coverage was obtained in 67% of the cases, while the planning CT was taken at the temporal mean tumor position, 56% at the end‐exhale position, and 11.1% at the end‐inhale position. Sixty‐six percent of the cases recorded with minimum target dose coverage, while the planning CT was taken either at the end‐inhale or end‐exhale phases.

**Table 3:** The mean conformity index (CI), the mean homogeneity index (HI), and the mean target coverage index (TGI) of the three phased‐specific dose plans. CI was evaluated at 99.5% target coverage. The amount of dose coverage as a function of planning CT taken at end‐exhale, end‐inhale, and mean tumor position is shown in the 5th to 7th columns

*Target Dose Coverage*		
Patient	*CI of 3 Dose Plans* (mean±SD)	*HI of 3 Dose Plans* (mean±SD)	*TC1 (%) of 3 Dose Plans* (mean±SD)	${\text{Vex}}_{20\text{Gy}}(\%)$	${\text{Vin}}_{20\text{Gy}}(\%)$	${\text{Vmean}}_{20\text{Gy}}(\%)$
1	1.80±0.04	1.21±0.01	99.7±0.1	57.4^b^	63.1	71.5^a^
2	1.95±0.18	1.22±0.02	99.8±0.2	85.2^b^	91.0	95.9^a^
3	1.86±0.04	1.20±0.01	99.5±0.0	80.0	73.0^b^	89.2^a^
4	1.65±0.04	1.19±0.00	99.8±0.0	97.7^a^	96.6	97.7^a^
5	1.84±0.04	1.22±0.01	99.8±0.0	94.6^a^	92.8	92.9
6	1.50±0.03	1.21±0.01	99.8±0.2	96.7^a^	90.7^b^	96.7^a^
7	1.67±0.05	1.20±0.01	99.6±0.1	99.1^a^	95.7	95.6
8	1.69±0.01	1.22±0.00	99.8±0.1	60.4^b^	83.2^a^	79.5
9	1.53±0.01	1.24±0.02	99.8±0.2	90.4^a^	66.9^b^	84.0

a ^a^ Indicates the maximum target dose coverage.

b ^b^ Indicates minimum coverage.

## IV. DISCUSSION

The efficacy of applying the “deform” dose along with deformable image registration in adaptive radiotherapy has long been debated due to the lack of comprehensive validation methodology in DIR.^(23)^ Validation of DIR's accuracy is difficult especially for clinical study because of the frequent lack of identifiable physical landmarks. Phantom‐based validation would however omit the factors that existed only clinically. Brock et al.^(24)^ assessed the accuracy of several underdeveloped DIR algorithms in 21 centers on a common set of patients’ data at the anatomical sites of lung, liver, and prostate. The study reported that all algorithms performed well for the cases of lung and liver, as the image contrast at those deformation fields was often consistent with stable breathing. However, artifacts caused by the presence of rectal gas and the substantial deformation of the prostate posed the challenge. The findings indicated that, no matter how meticulous the DIR algorithm is, the accuracy of DIR can be significantly affected by the characteristics of deformation field. Since IM, the algorithm of DIR employed in this study, is based on image intensity matching, stable CT images to improve spatial and contrast resolution of image are therefore considered important. Hence, efforts to select “reliable” images with minimal artifacts are a prerequisite and the primary means of maximizing registration accuracy. In this study, the image artifacts induced from abrupt breathing, which could significantly affect the image registration accuracy, were avoided by carefully selection of cases with stable breathing.

Despite there being no conclusion on the robustness of the DIR algorithms for advanced radiotherapy up to date, their contribution to improving the accuracy of dose distribution for deformable targets is evident. Studies^25^, ^26^, ^27^ have evaluated the performance of DIR registration and showed that deformable image registration not only improves the accuracy of image localization but also the accumulated target dose distribution as compared to the ridge‐based image registration. Janssens et al.^(27)^ verified the accuracy of DIR for 4D dose accumulation at deformation field based on phantom‐based dosimetric measurement. The difference between the estimated and measured doses was found significantly to decrease using DIR. Janssens et al. also reported that the maximum error in dose estimation was reduced from 96% to 3.5% with the DIR as compared to no registration. In this study, the efficacy of nonridge 4D dose calculation using DIR was evaluated, in which the target coverage as a function of phase‐specific planning CT was calculated. The results were found to be consistent with the literatures’ expectations. Studies^28^, ^29^, ^30^ have suggested if the tumor is irradiated at its average position during the respiratory cycle, optimal dose coverage would be obtained even if the tumor is not fully within the high‐dose region for a small part of the breathing cycle due to the presence of the wide‐beam penumbra. Moreover, dose planning performed at the end‐exhale phase was also suggested to maximize the target coverage with the most stable or reproducible target position. Both hypotheses were well verified in this study. As shown by the results of the study, in most of the cases (88.9%), planning CT taken at either the temporal mean tumor position or the end‐exhale position obtained the maximum target coverage. Moreover, the dose plan which obtained the maximum target coverage would also provide the best dose conformity as the dose volume of all the studied dose plans was meant to be equivalent (similar CI). In the past the ITV has often been defined in the space domain; the results of this study show that, if the temporal factor is also considered in defining the ITV, more optimum dose coverage can be obtained.

There were a few commercially available software products providing 4D dose calculation. The limitations of applications are often not addressed by the vendors. In this study, the limitations and complexity of the DIR for clinical implementation had been well attended to. Moreover, the 4D dose tool box is designed in several discrete levels, enabling quality assurances to be performed at different time phases for DIR and accumulated dose verification. The database is structured in “stack‐up” arrangement, permitting data generated from each individual process (e.g., IM, DE‐DOSE‐REG) to be assessed and confirmed via visual examination (e.g., AUTO‐CERR‐DOSE). The concise and systematic designs in data storage facilitate efficient and flexible data manipulation for 4D dose calculation. For instant, the voxel doses at each time phase are sequentially stored in a single array (${\text{TD}}_{X}$), thus allowing the accumulated target dose at any periods of the breathing cycle to be easily summated and analyzed. Thus, for the cases of respiratory‐gated treatment, the duty‐cycle can be effectively selected according to the estimated resultant dose calculated with the 4D tool box. The 4D tool box could be a useful tool to estimate the therapeutic gain for the selection of treatment technique.

## V. CONCLUSIONS

Accurate deformable image registration is often a challenging clinical problem to tackle; its limitations and applicability should be within our grasp before application. The efficacy of using the studied tool box for clinical application was confirmed with the retrospective studies of nine patients. The logistic and the robustness of the 4D tool box were shown to be feasible for nonridged 4D dose calculation. With the qualitative 4D CT images acquired under stable breathing, the studied tool box was proved to be useful and reliable for 4D dose calculation.

## ACKNOWLEDGMENTS

he authors are deeply grateful to Brainlab AG (Feldkirchen, Germany) for the retrieval of the morphed vector which relates the changes in morphology between different phases of the 4D CT. The authors would also like to thank Dr. T. V. How for fruitful discussions and Miss Chloe Lau for manuscript discussions.

## COPYRIGHT

This work is licensed under a Creative Commons Attribution 4.0 International License.
